# Physical activity level in Korean adults: the Korea National Health and Nutrition Examination Survey 2017

**DOI:** 10.4178/epih.e2019047

**Published:** 2019-11-09

**Authors:** Ki-Yong An

**Affiliations:** Faculty of Kinesiology, Sport, and Recreation, University of Alberta, Edmonton, AB, Canada

**Keywords:** Exercise, Health equity, Sedentary behavior, Health, Adult

## Abstract

**OBJECTIVES:**

This study investigated physical activity (PA) participation based on demographic, physical, and psychological variables in Korean adults.

**METHODS:**

Participants were divided into four groups (combined, aerobic only, resistance only, and neither) based on meeting the PA guidelines using moderate and vigorous PA time and resistance exercise frequency from the Korea National Health and Nutrition Examination Survey 2017. The association between meeting the PA guidelines and demographic, medical, fitness, lifestyle, and psychological variables were analyzed using complex samples crosstabs and a general linear model.

**RESULTS:**

Of the 5,820 Korean adults, 66.0% did not meet any of the guidelines. Among demographic factors, sex, age, marital status, income, education level, occupation, and employment status were associated with meeting the PA guidelines. Chronic disease prevalence, weight, waist circumference, body mass index, diastolic blood pressure, glucose, high-density lipoprotein and triglyceride levels, hand-grip strength, resting heart rate, and family history of chronic disease in the medical and fitness variables; frequency of drinking and eating breakfast, total calorie, water, protein, and fat intake in the lifestyle variables; and perceived stress, depression, suicidal thoughts, and quality of life in the psychological variables were associated with meeting PA guidelines.

**CONCLUSIONS:**

Most Korean adults participate in insufficient PA. Moreover, individuals who are socially underprivileged, have low-income or poor physical and mental health conditions participated in relatively less PA. Our findings suggest that government and individual efforts are required to increase PA and resolve health inequality in Korean adults.

## INTRODUCTION

Exercise and physical activity (PA) that prevent obesity and various chronic diseases and improve anxiety and depression, are cost-effective ways to enhance physical and mental health without any side effects [1,2]. The World Health Organization (WHO) has targeted the reduction in the prevalence of physical inactivity as one of the goals of global action plan 2013-2020 and is working to decrease the population of people with insufficient PA worldwide [3]. With the increasing importance of PA, the USA, Canada, Australia, and several European countries develop PA guidelines at the national level to encourage people to participate in more PA [4-8]. Additionally, there has been extensive research to investigate the PA level nationwide to increase people’s engagement in PAs [9-13]. Similarly, an increasing interest in PA in Korea has led to various studies on PA, but since most studies target populations with a specific age, sex or disease and/or include different variables, it is difficult to compare the results and the findings are different between studies. Therefore, it is necessary to examine the general PA level in Korea and the association between PA and various correlates using representative data. Furthermore, since PA is influenced by various environmental, cultural, and social factors [14-17], it should be consistently monitored as these factors change. Nevertheless, few studies [18,19] have reported the PA level in Korean using representative data.

Therefore, the present study aims to investigate the PA participation in Korean adults based on meeting the WHO PA guidelines according to socio-demographic, medical-related and health-related, lifestyle, and psychological variables using the Korea National Health and Nutrition Examination Survey (KNHANES) 2017 dataset.

## MATERIALS AND METHODS

### Data collection and participants

KNHANES is a nationwide health and nutrition survey conducted annually by trained specialists under the supervision of the Korea Centers for Disease Control and Prevention (KCDC). The survey consists of a health questionnaire survey, an examination survey, and a nutrition survey. The health questionnaire survey and examination surveys were conducted at mobile examination centers, while the nutrition survey was conducted by visiting the primary sampling unit in person. In the health questionnaire surveys, data for education, economic activity, disease prevalence, and healthcare utilization and the entire nutrition survey were collected by a face-to-face interview, and data for health behaviors including smoking and drinking were collected by self-reported questionnaires. Additionally, a health examination was conducted by direct measurement, observation, and specimen analysis. Of the total 8,127 participants of the 2017 survey, 5,820 adults aged 19 years and over who had responded to the PA survey were included in this study.

### Physical activity assessment

The PA level was measured using the Korean version of the modified Global Physical Activity Questionnaire (GPAQ) developed by the WHO, which includes the categories of weekly walking and resistance exercise added to the original GPAQ. The aerobic PA level was calculated as the sum of all PAs during leisure time and at work. To calculate the time spent on moderate-to-vigorous aerobic physical activity (MVPA) in minutes, we first converted the time spent weekly on vigorous aerobic activity into minutes, doubled the amount considering the metabolic equivalents according to activity intensity and then added the time spent weekly on moderate aerobic activity minutes. Resistance exercise frequency was measured as the number of days in a week. The subjects were divided into the following four groups based on their compliance with the WHO guidelines on aerobic PA and resistance exercise, recommended to both adults aged 18-64 years and the elderly aged over 65 years: (1) Aerobic only (≥150 min/wk of moderate aerobic activity, ≥75 min/wk of vigorous aerobic activity, or an equivalent combination of the two), (2) Resistance only (at least 2 d/wk of resistance exercises, (3) Combined (meeting both aerobic and resistance exercise guidelines), and (4) Neither (meeting neither guidelines).

### Assessment of correlates

We analyzed the subject’s compliance with the PA guidelines based on the socio-demographic, medical-related and health-related, lifestyle and psychological variables. The socio-demographic variables included sex, age, marital status, income, location, educational level, occupation, job status, a position at work, and employment status. The medical-related and health-related variables included chronic disease prevalence, cancer prevalence, weight, waist circumference (WC), body mass index (BMI), blood pressure, fasting glucose, total cholesterol (TC), high-density lipoprotein (HDL), triglyceride (TG) levels, hand-grip strength (the average of three measurements in the dominant hand) [20], resting heart rate (RHR), and family history of chronic disease. Lifestyle variables included smoking, drinking, sleep time, frequency of breakfast per week, total energy, water, carbohydrate, protein, and fat intake per day. Psychological variables included perceived stress, past history of depression lasting over two weeks, current depression prevalence, suicidal thoughts within the past year, past history of psychological counseling, and the five dimensions of health-related quality of life (HRQoL) such as mobility, self-care, usual activity, pain/discomfort, and anxiety/depression.

### Statistical analysis

All statistical analyses were conducted using a complex sample analysis. We used the Complex Samples Crosstabs to analyze the relationship between correlates and compliance with PA guidelines for nominal variables and the complex samples general linear model (CSGLM) for continuous variables. In addition, we also used the CSGLM to analyze the group differences in moderate-to-vigorous PA time and resistance exercise frequency for each variable. For nominal variables with uneven distribution, we redefined the categories for analysis (e.g., marital status: not married vs. living together after marriage vs. separated/widowed/divorced after marriage; HRQoL: no problem vs. any problems). All analyses were conducted using SPSS version 25 (IBM Corp., Armonk, NY, USA).

### Ethics statement

KNHANES has been conducting without approval by the research ethics review committee because it is considered the research for public welfare which is conducted by the Korean government since 2015.

## RESULTS

Table 1 shows the association between compliance with the PA guidelines and the socio-demographic variables. Of the total participants, most were in the neither group (66.0%), followed by resistance only (15.7%), combined (9.8%), and aerobic only groups (8.5%). The subjects’ compliance with the PA guidelines showed a significant correlation with sex (p<0.001), age (p<0.001), marital status (p<0.001), total household income (p<0.001), education level (p<0.001), occupation (p<0.001), and employment status (p=0.010) among the socio-demographic variables. In particular, among females aged over 65 years, those living without their spouse after marriage, in the lowest income group and education level, and had labor job demonstrated the lowest level of MVPA and resistance exercise frequency, also contingent workers tended to have lower MVPA level than regular workers.

Table 2 shows the association between compliance with the PA guidelines and the medical-related and health-related variables. The subjects’ compliance with the PA guidelines was significantly associated with chronic disease (p<0.001), weight (p<0.001), WC (p=0.005), BMI (p=0.001), diastolic blood pressure (p<0.001), fasting glucose levels (p<0.001), HDL levels (p=0.001), TG levels (p<0.001), hand-grip strength (p<0.001), RHR (p<0.001), and family history of chronic disease (p=0.001). The MVPA level was relatively lower for those with ≥2 chronic diseases, ≥1 cancer type, and a WC ≥90 cm for males and ≥80 cm for females. On the other hand, participants in the obesity group (BMI≥25.0 kg/m^2^) compared to the underweight group (BMI<18.5 kg/m^2^) or those with a family history of chronic disease, showed higher MVPA levels. In addition, those with a WC of ≥90 cm for males and ≥80 cm for females compared to normal WC or a BMI≥25.0 kg/m^2^ compared to the normal or overweight groups participated in resistance exercise relatively less.

Table 3 shows the subjects’ compliance with the PA guidelines in association with their lifestyle variables. The subjects’ compliance with the PA guidelines showed a significant correlation with drinking (p<0.001) and daily intake of total energy (p<0.001), water (p<0.001), protein (p<0.001), and fat (p<0.001). The MVPA and resistance exercise levels were higher for those drinking more than twice a week than for those who have not been drinking in the past year.

Table 4 shows the subjects’ compliance with the PA guidelines in association with HRQoL and psychological variables. The subjects’ compliance with the PA guidelines showed a significant correlation with perceived stress (p=0.015), past history of depression lasting over two weeks (p<0.001), suicidal thoughts within one year (p=0.026), and HRQoL for mobility (p<0.001), self-care (p<0.001), daily activity (p<0.001), pain/discomfort (p<0.001), and anxiety/depression (p=0.002). Moreover, those with the highest stress levels, past history of depression lasting over two weeks, current depression, suicidal thoughts within one year and any problems with mobility, self-care, pain/discomfort, and anxiety/depression had lower levels of MVPA than others. The level of resistance exercise frequency was lower for those with the highest stress levels, depression lasting over two weeks, suicidal thoughts within a year and problems with mobility, self-care, daily activity, pain/discomfort, and anxiety/depression than those without.

## DISCUSSION

This study analyzed the Korean adults’ compliance with PA guidelines in association with the socio-demographic, medical-related and health-related, lifestyle, and psychological variables. The results revealed several characteristics of PA participation among Korean adults. First, the Korean adults demonstrated very low compliance with the PA guidelines. Of the total participants analyzed, 66.0% failed to comply with neither aerobic exercise nor resistance exercise. According to Yang [19], the proportion of Korean adults with insufficient PA was 24.6% in 2008, which continued to increase and reached 42.9% by 2014. This is lower than the proportion of physically inactive adults worldwide reported in 2012, which was 31.1% [21]. Given the various benefits of PA on physical and mental health improvement [1,2], the government’s efforts are urgently required to encourage PA and create an environment for PA participation. Once the PA level increases, then it will not only improve public health but also reduce the costs of medical care.

Second, the PA level in Korean adults significantly differed depending on health inequality factors. Older females with lower income and education levels, in other words, a minority group, showed relatively lower compliance with the PA guidelines. These results imply that socioeconomic factors, as well as physiological factors, have a significant impact on healthy behavior. The difference in PA levels induced by such socioeconomic factors can trigger a vicious cycle of health inequality. Insufficient PA leads to chronic illnesses and weakened physical/mental health, which in turn leads to increased medical costs and limited socioeconomic activities due to illness that can easily circle back to physical inactivity and health inequality. To end this cycle, we need substantial and specific solutions like providing free education on PA in schools and health centers or media-based campaigns promoting PA participation.

Third, participants with a high risk of chronic diseases and poor mental health had lower PA levels than healthier participants, even though they needed PA more. Since the present study is a cross-sectional study, the causality between PA and the various correlates cannot be identified. In other words, it is possible either poor physical/mental health could cause physical inactivity or vice versa. In this study, however, we clarified the findings of previous studies that there is a significant relationship between PA and various chronic diseases [22,23], as well as mental health [24,25]. With the collective effort of medical professionals in emphasizing the importance of PA and recommending the appropriate level and method of PA in medical institutions, the PA levels of the Korean adults could be markedly increased.

Furthermore, the results of this study showed that the PA level was low not only in the obesity group but also in the underweight group. Previous studies have reported that both underweight and obese people have relatively higher mortality rates than people with normal weight [26,27]. Recently, people in Korea tend to prefer a skinny body shape and attempt extreme diets to reduce weight using unhealthy methods. To maintain good health conditions, efforts to increase PA levels must be accompanied by efforts to maintain healthy body weight.

Additionally, our findings are consistent with previous studies [28,29] that reported that physical fitness is associated with PA levels. Hand-grip strength is the only fitness factor in the KNHANES. There are a few factors related to lung function but they are inappropriate to present cardiopulmonary fitness and the abundance of missing values makes them difficult to be used for this study. RHR, another fitness-related factor used in this study is directly linked to aerobic exercise. Although we cannot deem RHR as an aerobic fitness factor without considering the age and fitness level of an individual, the fact that aerobic exercise increases cardiac output and decreases RHR suggests that it is possible to indirectly predict aerobic fitness by RHR. Moreover, since RHR is closely related to risk factors of various chronic diseases such as diabetes, cardiovascular diseases, and metabolic syndrome [30-32], it can act as an important health variable by itself. As physical fitness is highly linked to many health risks, the inclusion of aerobic fitness factors in the KNHANES will greatly help to monitor and examine the physical health of the Korean people.

Furthermore, we identified that a family history of chronic disease, drinking, and diet-related variables were associated with PA participation. Surprisingly, however, people with a family history of chronic disease, heavy drinking, and high-calorie intake tended to participate in more PA. It can be explained by the fact that a family history of chronic disease may motivate exercise, males tend to be heavier drinkers than females, and the overall energy intake in Korean adults is not that high. Nevertheless, further research and various statistical analyses are required to accurately interpret these relationships.

A limitation of this study is that it is a cross-sectional study with limited assessment variables (health-related fitness and medical variables). Our study, however, can provide basic data for PA research in Korean adults with its large sample size, analysis of correlations with various classifications of variables, the distinction between aerobic and resistance exercise guidelines, and most importantly, analysis of the latest data showing the overall PA participation among Korean adults.

In conclusion, this study investigated the PA levels in Korean adults in association with various correlates, which revealed that Korean adults participate in definitely low levels of PA. Moreover, PA engagement was not only associated with physiological factors such as sex, age, lifestyle, physical health-related variables, but also with diverse socioeconomic and environmental factors including health inequality-related factors. To improve public health in Korea, we need both the collective effort of the government, educational, and medical institutions and individual efforts to recognize the importance of PA and its practice.

## Figures and Tables

**Table 1.** Association between PA and socio-demographic variables in Korean adults

Variables	%	Neither	Resistance only	Aerobic only	Combined	p-value	PA^1^	
							MV (min/wk)	Resistance (d/wk)
Total		4,021 [26,136,896 (66.0)]	880 [6,214,780 (15.7)]	436 [3,365,609 (8.5)]	483 [3,893,229 (9.8)]		83.5±4.0	0.82±0.03
Sex								
Male	49.6	1,532 [11,363,150 (57.9)]	521 [3,819,234 (19.4)]	216 [1,846,260 (9.4)]	299 [2,612,582 (13.3)]	<0.001	109.5±6.6^1^	1.09±0.04^1^
Female (reference)	50.4	2,489 [14,773,746 (74.0)]	359 [2,395,546 (12.0)]	220 [1,519,349 (7.6)]	184 [1,280,647 (6.4)]		57.9±3.7	0.56±0.03
Age (yr)								
19-44	45.4	1,306 [11,022,509 (61.3)]	313 [2,732,177 (15.2)]	204 [1,802,384 (10.0)]	256 [2,419,007 (13.5)]	<0.001	111.7±6.6^1^	0.86±0.04^1^
45-64	38.1	1,513 [9,964,925 (66.0)]	353 [2,473,339 (16.4)]	189 [1,372,764 (9.1)]	181 [1,285,525 (8.5)]		72.7±4.7^1^	0.82±0.04
≥65 (reference)	16.5	1,201 [5,149,462 (78.8)]	214 [1,009,263 (15.4)]	43 [190,460 (2.9)]	46 [188,697 (2.9)]		30.9±4.9	0.71±0.05
Marital status								
Not married	23.8	585 [5,365,207 (56.9)]	170 [1,635,760 (17.3)]	89 [907,329 (9.6)]	151 [1,521,406 (16.1)]	<0.001	126.6±10.4^1^	1.04±0.06^1^
Married/live together	65.5	2,746 [17,288,916 (66.7)]	633 [4,142,231 (16.0)]	319 [2,291,515 (8.8)]	303 [2,197,358 (8.5)]		76.1±4.3^1^	0.79±0.03^1^
Married/live separately (reference)	10.7	688 [3,469,540 (81.7)]	77 [436,789 (10.3)]	28 [166,765 (3.9)]	29 [174,465 (4.1)]		33.5±5.0	0.52±0.05
Income								
High	32.0	1,021 [7,396,851 (58.6)]	258 [1,962,602 (25.6)]	186 [1,509,455 (12.0)]	220 [1,745,228 (13.8)]	<0.001	115.0±6.7^1^	0.93±0.04^1^
Mid-high	29.7	1,054 [7,613,712 (65.0)]	265 [1,926,915 (16.4)]	135 [1,003,437 (8.6)]	134 [1,174,657 (10.0)]		85.2±7.1^1^	0.84±0.04^1^
Mid-low	23.0	1,005 [6,309,395 (69.5)]	213 [1,459,465 (16.1)]	82 [620,262 (6.8)]	94 [688,379 (7.6)]		65.8±7.9	0.77±0.05
Low (reference)	15.3	929 [4,718,458 (78.0)]	142 [843,942 (13.9)]	32 [220,666 (3.6)]	34 [269,728 (4.5)]		41.7±9.8	0.64±0.08
Location								
Urban	85.0	3,190 [21,891,690 (65.0)]	761 [5,391,430 (16.0)]	381 [2,933,863 (8.7)]	436 [3,447,515 (10.2)]	0.143	83.6±4.2	0.83±0.03
Rural (reference)	15.0	831 [4,245,205 (71.4)]	119 [823,350 (13.8)]	55 [431,745 (7.3)]	47 [445,714 (7.5)]		82.8±13.4	0.77±0.10
Education level								
University/college	43.6	1,315 [10,021,785 (58.1)]	390 [3,007,345 (17.4)]	242 [2,009,433 (11.6)]	263 [2,213,219 (12.8)]	<0.001	107.8±6.2^1^	0.91±0.04^1^
High school	33.0	1,173 [8,459,210 (64.9)]	289 [2,105,752 (16.1)]	141 [1,088,624 (8.3)]	174 [1,389,891 (10.7)]		88.8±6.6^1^	0.88±0.05^1^
Middle school	8.8	437 [2,565,852 (74.0)]	95 [565,882 (16.3)]	31 [163,266 (4.7)]	23 [173,569 (5.0)]		56.6±17.5^1^	0.84±0.10^1^
Elementary school (reference)	14.6	1,088 [5,037,117 (87.1)]	105 [527,500 (9.1)]	22 [104,285 (1.8)]	23 [116,549 (2.0)]		16.0±2.5	0.42±0.04
Occupation								
Manager/profession	17.1	511 [3,927,068 (58.3)]	152 [1,231,194 (18.3)]	82 [724,036 (10.7)]	106 [859,419 (12.7)]	<0.001	107.3±9.3^1^	0.94±0.06^1^
Office job	12.9	404 [3,066,667 (60.3)]	114 [877,095 (17.3)]	67 [554,935 (10.9)]	70 [585,138 (11.5)]		93.1±8.7^1^	0.88±0.08^1^
Inoccupation (housewife/students)	35.1	1,623 [9,293,231 (67.0)]	334 [2,110,621 (15.2)]	145 [1,088,174 (7.8)]	179 [1,376,645 (9.9)]		79.8±6.7^1^	0.85±0.05^1^
Service industry	12.0	452 [3,124,938 (65.8)]	101 [764,866 (16.1)]	55 [394,778 (8.3)]	52 [464,145 (9.8)]		78.8±10.4	0.82±0.08^1^
Engineer	11.8	398 [3,195,767 (68.6)]	96 [731,258 (15.7)]	42 [322,911 (6.9)]	46 [410,697 (8.8)]		75.7±12.1	0.78±0.08^1^
Agriculture/forestry/fishery	3.2	237 [1,011,866 (79.0)]	27 [177,091 (13.8)]	11 [58,774 (4.6)]	4 [33,708 (2.6)]		55.3±22.3	0.49±0.08
Labor (reference)	7.9	389 [2,451,315 (78.7)]	55 [309,273 (9.9)]	32 [196,644 (6.3)]	25 [155,998 (5.0)]		54.3±9.4	0.53±0.06
Job status								
Employed	65.0	2,393 [16,805,826 (65.4)]	546 [4,104,159 (16.0)]	291 [2,277,435 (8.9)]	304 [2,516,584 (9.8)]	0.582	85.6±4.9	0.80±0.03
Unemployed (reference)	35.0	1,623 [9,293,231 (67.0)]	334 [2,110,621 (15.2)]	145 [1,088,174 (7.8)]	179 [1,376,645 (9.9)]		79.8±6.7	0.85±0.05
Position in work								
Employer/self-ownership	23.1	593 [3,827,031 (64.5)]	158 [1,112,280 (18.8)]	76 [523,433 (8.8)]	62 [468,944 (7.9)]	0.069	73.1±6.9^1^	0.87±0.06
Unpaid family worker	3.9	148 [757,704 (75.2)]	21 [139,975 (13.9)]	11 [65,065 (6.5)]	7 [44,828 (4.4)]		56.6±21.7	0.59±0.16
Employee (reference)	73.0	1,652 [12,221,091 (65.1)]	367 [2,851,904 (15.2)]	204 [1,688,936 (9.0)]	235 [2,002,812 (10.7)]		91.1±5.8	0.79±0.04
Employment status	51.5							
Regular (permanent) worker		739 [5,975,330 (61.9)]	181 [1,515,160 (15.7)]	120 [1,009,134 (10.5)]	135 [1,155,242 (12.0)]	0.010	103.2±8.2^1^	0.81±0.05
Contingent worker (reference)	48.5	913 [6,245,760 (68.6)]	186 [1,336,744 (14.7)]	84 [679,802 (7.5)]	100 [847,570 (9.3)]		78.3±7.4	0.78±0.05

Values are presented as unweighted number [estimated number (%)] or mean±standard deviation. PA, physical activity; MV, moderate to vigorous aerobic.

1 A p-value was calculated versus reference group.

* p<0.05.

**Table 2.** Association between PA and medical- and health-related variables in Korean adults

Variables	%	Neither	Resistance only^1^	Aerobic only^1^	Combined^1^	p-value	PA^1^	
							MV (min/wk)	Resistance (d/wk)
Chronic disease (n)^2^								
None	67.6	2,289 [17,014,375 (63.6)]	526 [4,052,075 (15.1)]	314 [2,546,704 (9.5)]	368 [3,149,127 (11.8)]	<0.001	97.1±5.0^*^	0.84±0.03
1	17.5	852 [4,760,985 (68.5)]	188 [1,204,742 (17.3)]	70 [491,257 (7.1)]	77 [492,554 (7.1)]		65.6±5.8^*^	0.82±0.05
≥2 (reference)	14.9	880 [4,361,535 (73.9)]	166 [957,962 (16.2)]	52 [327,648 (5.6)]	38 [251,548 (4.3)]		43.1±6.3	0.73±0.06
Cancer								
None	98.1	3,925 [25,592,704 (65.8)]	861 [6,113,361 (15.7)]	428 [3,321,728 (8.5)]	474 [3,847,068 (9.9)]	0.310	84.1±4.1^*^	0.82±0.03
≥1 (reference)	1.9	96 [544,192 (74.0)]	19 [101,418 (13.8)]	8 [43,881 (6.0)]	9 [46,161 (6.3)]		52.0±11.7	0.73±0.15
Weight (kg)	-	64.0±0.3	66.0±0.5^*^	69.0±0.8^*^	68.0±0.7^*^	<0.001	NA	NA
Waist circumference (cm)^3^		81.9±0.3	81.7±0.4	82.6±0.6	80.1±0.6^*^	0.005	NA	NA
Normal	64.8	2,324 [15,815,041 (61.9)]	607 [4,372,279 (17.1)]	276 [2,196,212 (8.6)]	384 [3,152,120 (12.3)]	<0.001	95.8±5.6^*^	0.95±0.03^*^
Obesity (reference)	35.2	1,673 [10,152,017 (73.3)]	270 [1,821,628 (13.1)]	159 [1,165,309 (8.4)]	97 [719,334 (5.2)]		61.5±4.4	0.58±0.03
BMI (kg/m^2^)^4^		23.9±0.1	23.7±0.1	24.6±0.2^*^	23.7±0.2	0.001	NA	NA
Underweight	4.2	173 [1,238,618 (75.7)]	29 [207,971 (12.7)]	6 [43,594 (2.7)]	19 [145,125 (8.9)]	<0.001	52.3±11.4^*^	0.64±0.12
Normal weight	38.7	1,531 [10,038,066 (66.0)]	360 [2,533,130 (16.7)]	147 [1,139,104 (7.5)]	192 [1,499,179 (9.9)]		74.0±5.5	0.84±0.04^*^
Overweight	22.6	880 [5,483,400 (61.5)]	215 [1,570,909 (17.6)]	97 [755,233 (8.5)]	129 [1,101,358 (12.4)]		105.4±10.1	1.03±0.06^*^
Obesity (reference)	34.5	1,408 [9,153,730 (67.4)]	275 [1,894,259 (13.9)]	185 [1,420,723 (10.5)]	141 [1,118,727 (8.2)]		84.5±6.1	0.69±0.04
Blood pressure (mmHg)								
SBP	-	117.6±0.4	117.1±0.7	116.8±0.8	115.4±0.7^*^	0.065	NA	NA
DBP	-	75.2±0.2	76.2±0.5^*^	77.8±0.5^*^	75.9±0.5	<0.001	NA	NA
Blood profile (mg/dL)								
Glucose	-	100.7±0.5	99.3±0.7	97.3±0.9^*^	95.3±0.7^*^	<0.001	NA	NA
TC	-	194.1±0.8	191.0±1.6	196.1±2.3	191.6±1.8	0.135	NA	NA
HDL	-	50.9±0.2	51.4±0.4	50.8±0.8	53.4±0.6^*^	0.001	NA	NA
TG	-	137.1±2.3	133.4±4.5	149.9±7.2	117.6±4.6^*^	<0.001	NA	NA
Hand-grip strength (kg)^5^	-	28.4±0.2	32.6±0.4^*^	32.1±0.6^*^	35.4±0.6^*^	<0.001	NA	NA
RHR (beat/min)	-	70.7±0.2	70.1±0.4	68.8±0.5^*^	67.8±0.6^*^	<0.001	NA	NA
Family history								
No	41.2	1,757 [11,352,364 (69.6)]	343 [2,337,394 (14.3)]	144 [1,123,515 (6.9)]	186 [1,489,851 (9.1)]	0.001	74.3±5.0^*^	0.79±0.04
Yes (reference)	58.8	2,258 [14,756,927 (63.4)]	536 [3,872,066 (16.6)]	291 [2,238,249 (9.6)]	296 [2,393,505 (10.3)]		90.0±5.4	0.84±0.03

Values are presented as unweighted number [estimated number (%)] or mean±standard deviation. PA, physical activity; MV, moderate to vigorous aerobic; BMI, body mass index; SBP, systolic blood pressure; DBP, diastolic blood pressure; TC, total cholesterol; HDL, high-density lipoprotein; TG, triglyceride; RHR, resting heart rate; NA, not applicable.

1 A p-value was calculated versus neither or reference group.

2 Chronic disease was counted except for cancer.

3 Waist circumference: obesity ≥90 cm for males and ≥80 cm for females.

4 BMI (kg/m^2^): underweight <18.5, 18.5≤ normal<23.0, 23.0≤overweight<25.0, obesity≥25.0.

5 Hand-grip strength values are the average of three measurements for the dominant hand (higher value is used for ambidextrous participants).

* p<0.05.

**Table 3.** Association between PA and lifestyle variables in Korean adults

Variables	%	Neither	Resistance only^1^	Aerobic only^1^	Combined^1^	p-value	PA^1^	
							MV (min/wk)	Resistance (d/wk)
Smoking								
No	79.1	3,301 [20,446,756 (65.2)]	723 [5,016,478 (16.0)]	365 [2,760,063 (8.8)]	402 [3,116,831 (9.9)]	0.294	84.5±4.5	0.83±0.03
Yes (reference)	20.9	720 [5,690,140 (68.8)]	157 [1,198,301 (14.5)]	71 [605,546 (7.3)]	81 [776,398 (9.4)]		79.9±8.4	0.77±0.06
Drink								
Never/no within 1 yr	22.7	1,228 [6,710,410 (74.5)]	216 [1,334,565 (14.8)]	78 [517,954 (5.7)]	61 [446,184 (5.0)]	<0.001	50.8±7.2^*^	0.64±0.05^*^
≤1/wk	53.0	1,936 [13,291,122 (63.3)]	453 [3,325,489 (15.8)]	244 [1,898,214 (9.0)]	299 [2,471,272 (11.8)]		93.3±5.2	0.87±0.03
≥2/wk (reference)	24.3	857 [6,135,364 (63.8)]	211 [1,554,726 (16.2)]	114 [949,440 (9.9)]	123 [975,773 (10.1)]		92.9±8.0	0.88±0.06
Sleeping time (hr)								
≥7	63.3	2,544 [16,672,394 (66.7)]	533 [3,788,823 (15.2)]	274 [2,171,065 (8.7)]	291 [2,362,545 (9.5)]	0.326	80.8±4.9	0.80±0.03
<7 (reference)	36.7	1,456 [9,372,539 (64.6)]	346 [2,421,614 (16.7)]	161 [1,190,699 (8.2)]	191 [1,525,436 (10.5)]		88.6±6.0	0.86±0.04
Frequency of breakfast (d/wk)^2^								
≥5	59.6	2,438 [15,872,878 (66.7)]	519 [3,874,866 (16.3)]	232 [1,835,349 (7.7)]	248 [2,212,135 (9.3)]	0.174	75.9±4.7	0.85±0.04
<5 (reference)	40.4	1,131 [10,384,185 (64.4)]	247 [2,503,065 (15.5)]	145 [1,536,873 (9.5)]	150 [1,689,054 (10.5)]		89.9±7.0	0.79±0.04
Energy intake (kcal/d)^2^	-	1,892.1±21.5	1,991.2±42.1^*^	2,028.2±51.7^*^	2,122.6±60.0^*^	<0.001	NA	NA
Water intake (g/d)^2^	-	1,053.1±17.1	1,149.5±35.7^*^	1,232.9±31.3^*^	1,303.5±37.0^*^	<0.001	NA	NA
Carbohydrate intake (g/d)^2^	-	294.9±3.0	306.2±5.6^*^	304.1±6.2	301.6±7.8	0.120	NA	NA
Protein intake (g/d)^2^	-	70.5±1.0	75.3±2.2^*^	77.2±2.2^*^	85.9±3.0^*^	<0.001	NA	NA
Fat intake (g/d)^2^	-	44.4±0.9	47.7±1.4^*^	53.8±2.7^*^	57.2±2.7^*^	<0.001	NA	NA

Values are presented as unweighted number [estimated number (%)] or mean±standard deviation.

PA, physical activity; MV, moderate to vigorous aerobic; NA, not applicable.

1 A p-value was calculated versus neither or reference group.

2 Nutrition survey weighted data.

* p<0.05.

**Table 4.** Association between PA and psychological variables in Korean adults

Variables			%	Neither	Resistance only	Aerobic only	Combined	p-value	PA^1^	
									MV (min/wk)	Resistance (d/wk)
Perceived stress										
	Barely		13.9	655 [3,660,732 (66.7)]	151 [902,023 (16.4)]	61 [406,281 (7.4)]	74 [521,236 (9.5)]	0.015	77.4±8.6^*^	0.93±0.07^*^
	A little		57.1	2,196 [14,384,886 (63.7)]	520 [3,711,842 (16.4)]	260 [2,006,379 (8.9)]	300 [2,469,255 (10.9)]		93.3±5.5^*^	0.90±0.03^*^
	Much		24.5	951 [6,680,008 (68.9)]	180 [1,368,591 (14.1)]	101 [842,758 (8.7)]	97 [810,647 (8.4)]		71.8±5.4^*^	0.65±0.05
	Very much (reference)		4.4	208 [1,349,238 (76.8)]	27 [212,170 (12.1)]	14 [110,190 (6.3)]	11 [85,222 (4.9)]		43.7±7.7	0.46±0.10
Depression over 2 wk										
	No		88.3	3,444 [22,616,189 (64.8)]	797 [5,621,820 (16.1)]	402 [3,106,487 (8.9)]	439 [3,547,657 (10.2)]	<0.001	86.3±4.3^*^	0.85±0.03^*^
	Yes (reference)		11.7	568 [3,467,406 (74.8)]	81 [572,806 (12.4)]	34 [259,121 (5.6)]	43 [338,703 (7.3)]		63.1±9.1	0.56±0.05
Current depression										
	No		97.9	3,902 [25,527,907 (65.9)]	856 [6,061,816 (15.6)]	431 [3,338,864 (8.6)]	478 [3,836,926 (9.9)]	0.170	84.5±4.1^*^	0.82±0.03
	Yes (reference)		2.1	119 [608,989 (72.1)]	24 [152,964 (18.1)]	5 [26,745 (3.2)]	5 [56,303 (6.7)]		39.5±13.1	0.77±0.15
Thinking suicide for a year										
	No		95.3	3,782 [24,679,629 (65.5)]	841 [5,928,594 (15.7)]	421 [3,262,604 (8.7)]	474 [3,802,811 (10.1)]	0.026	85.5±4.2^*^	0.83±0.02^*^
	Yes (reference)		4.7	230 [1,403,966 (75.6)]	37 [266,032 (14.3)]	15 [103,005 (5.5)]	8 [83,549 (4.5)]		44.8±12.8	0.57±0.11
Psychological counselling for a year										
	No		97.2	3,892 [25,359,835 (66.0)]	848 [5,992,457 (15.6)]	424 [3,288,442 (8.6)]	471 [3,795,700 (9.9)	0.710	84.3±4.1	0.82±0.03
	Yes (reference)		2.8	122 [731,273 (66.4)]	30 [202,169 (18.4)]	12 [77,167 (7.0)]	11 [90,660 (8.2)]		57.8±14.3	0.76±0.12
HRQoL										
	Mobility									
		No problem	89.9	3,335 [22,831,301 (64.1)]	792 [5,717,456 (16.1)]	418 [3,285,001 (9.2)]	464 [3,781,737 (10.6)]	<0.001	89.5±4.3^*^	0.85±0.03^*^
		Any problem (reference)	10.1	686 [3,305,595 (82.7)]	88 [497,323 (12.4)]	18 [80,608 (2.0)]	19 [111,492 (2.8)]		30.5±10.4	0.53±0.06
	Self-care									
		No problem	97.3	3,831 [25,204,176 (65.4)]	861 [6,112,534 (15.9)]	432 [3,335,734 (8.7)]	478 [3,869,807 (10.0)]	<0.001	85.2±4.1^*^	0.83±0.03^*^
		Any problems (reference)	2.7	190 [932,719 (85.7)]	19 [102,246 (9.4)]	4 [29,875 (2.7)]	5 [23,422 (2.2)]		24.4±12.8	0.38±0.08
	Usual activity									
		No problem	94.4	3,654 [24,352,985 (65.1)]	832 [5,963,909 (15.9)]	425 [3,267,270 (8.7)]	471 [3,813,986 (10.2)]	<0.001	85.5±4.2	0.84±0.03^*^
		Any problems (reference)	5.6	366 [1,778,745 (80.6)]	48 [250,870 (11.4)]	11 [98,339 (4.5)]	12 [79,243 (3.6)]		49.6±18.8	0.53±0.08
	Pain/discomfort									
		No problem	79.1	2,970 [20,164,281 (64.4)]	691 [4,946,862 (15.8)]	353 [2,754,421 (8.8)]	420 [3,462,990 (11.1)]	<0.001	89.6±4.5^*^	0.86±0.03^*^
		Any problems (reference)	20.9	1,050 [5,969,824 (72.1)]	189 [1,267,918 (15.3)]	83 [611,188 (7.4)]	63 [430,239 (5.2)]		60.7±6.8	0.68±0.05
	Anxiety/depression									
		No problem	92.9	3,634 [24,027,069 (65.3)]	813 [5,798,007 (15.8)]	411 [3,202,848 (8.7)]	464 [3,746,384 (10.2)]	0.002	86.8±4.3^*^	0.84±0.03^*^
		Any problems (reference)	7.1	384 [2,104,134 (74.3)]	67 [416,773 (14.7)]	25 [162,760 (5.8)]	19 [146,845 (5.2)]		40.6±5.5	0.63±0.08

Values are presented as unweighted number [estimated number (%)] or mean±standard deviation.

PA, physical activity; MV, moderate to vigorous aerobic; HRQoL, health-related quality of life.

1 A p-value was calculated versus reference group.

* p<0.05.

## SUPPLEMENTARY MATERIALS

Korean version is available at http://www.e-epih.org/.
