# Supplementary material for: Physical activity level in Korean adults: the Korea National Health and Nutrition Examination Survey 2017
Source: Epidemiol Health. 2019 Nov 9;41:e2019047. doi: 10.4178/epih.e2019047 (PMC6928466; doi:10.4178/epih.e2019047)
Supplement: Supplementary file 1 [file epih-41-e2019047-supplementary.pdf]

Volume: 41, Article ID: e2019047
https://doi.org/10.4178/epih.e2019047

한국 성인들의 신체활동 실천율 분석: 2017 년 국민 건강영양 조사 자료를 바탕으로

안기용

Physical activity level in Korean adults: the Korea National Health and Nutrition Examination Survey 2017

Ki-Yong An

University of Alberta

Corresponding author: Ki-Yong An

1-115 University Hall, University of Alberta, 116St. and 85Ave., Edmonton, Alberta, Canada T6G 2R3,

e-mail: kiyong1@ualberta.ca

-ABSTRACT-

OBJECTIVES: This study investigated physical activity (PA) participation based on demographic, physical, and psychological variables in Korean adults.

METHODS: Participants were divided into four groups (combined, aerobic only, resistance only, and neither) based on meeting the PA guidelines using moderate and vigorous PA time and resistance exercise frequency from the Korea National Health and Nutrition Examination Survey 2017. The association between meeting the PA guidelines and demographic, medical, fitness, lifestyle, and psychological variables were analyzed using complex samples crosstabs and a general linear model.

RESULTS: Of the 5,820 Korean adults, 66.0% did not meet any of the guidelines. Among demographic factors, sex, age, marital status, income, education level, occupation, and employment status were associated with meeting the PA guidelines. Chronic disease prevalence, weight, waist circumference, body mass index, diastolic blood pressure, glucose, high-density lipoprotein and triglyceride levels, hand-grip strength, resting heart rate, and family history of chronic disease in the medical and fitness variables; frequency of drinking and eating breakfast, total calorie, water, protein, and fat intake in the lifestyle variables; and perceived stress, depression, suicidal thoughts, and quality of life in the psychological variables were associated with meeting PA guidelines.

CONCLUSIONS: Most Korean adults participate in insufficient PA. Moreover, individuals who are socially underprivileged, have low-income or poor physical and mental health conditions participated in relatively less PA. Our findings suggest that government and individual efforts are required to increase PA and resolve health inequality in Korean adults.

KEY WORDS: Exercise, Health equity, Sedentary behavior, Health, Adult

INTRODUCTION

운동과 신체활동은 비만과 다양한 만성질환을 예방하고, 불안과 우울을 해소해 주는 등 부작용 없이 경제적으로 신체적, 정신적 건강을 증진시킬 수 있는 좋은 방법 중 하나이다 [1, 2]. 세계보건기구에서는 global action plan 2013-2020 의 목표 중 하나로 신체비활동 인구의 감소를 설정하고, 전세계적으로 불충분한 신체활동을 하는 인구를 줄이는데 힘쓰고 있다 [3]. 신체활동의 중요성이 강조됨에 따라 미국, 캐나다, 호주 및 유럽 국가들에서는 국가 차원에서 신체활동 관련 지침을 개발하여 국민들에게 적극적으로 신체활동을 권고하고 있으며 [4-8],

Volume: 41, Article ID: e2019047
https://doi.org/10.4178/epih.e2019047

신체활동 참여를 늘리기 위해 자국민들의 신체활동량을 조사, 분석하는 연구들이 활발히 진행되고 있다 [9-13]. 한국에서도 신체활동에 대한 관심이 점차 증가하면서 신체활동과 관련된 다양한 연구들이 발표되고 있지만, 대부분의 연구들이 특정 연령이나 성별, 또는 질환을 갖고 있는 인구집단을 대상으로 하거나 연구마다 각기 다른 변수들을 포함하고 있어 서로 비교가 어렵고, 연구결과가 상이한 경우도 많다. 따라서, 대표성이 있는 자료를 이용하여 한국인들이 신체활동을 얼마나 하고 있는지, 어떠한 요인들이 신체활동 참여에 영향을 미치는지에 대한 연구가 필요하다. 게다가 신체활동은 다양한 환경적, 문화적, 사회적 변인들의 영향을 받기 때문에 [14-17], 이러한 변인들의 변화에 따라 지속적으로 관찰되어야 한다. 그럼에도 불구하고, 대표성 있는 자료를 이용하여 한국인들의 신체활동량을 보고한 연구는 많지 않다 [18, 19].

따라서, 본 연구에서는 국민건강영양조사 최신자료인 2017 년 자료를 이용하여 세계보건기구에서 권장하는 신체활동 지침충족 여부에 따라 한국 성인들의 인구사회학적, 체력 및 의학적, 생활습관, 정신건강 변인에 따른 신체활동 실천율을 조사하였다.

METHODS

Data collection and participants

국민건강 영양조사는 매년 질병관리본부의 감독하에 전문조사원들에 의해 실시되는 전국 규모의 건강 및 영양 조사이다. 조사는 건강설문조사, 검진조사, 영양조사로 이루어지며, 건강설문조사와 검진조사는 이동검진센터에서, 영양조사는 대상 조사구를 직접 방문하여 실시되었다. 본 연구에 사용된 건강설문조사의 교육 및 경제활동, 이환, 의료이용 항목, 영양조사의 전체항목은 면접방법으로, 흡연, 음주 등 건강행태 영역은 자기기입식으로, 검진조사는 직접 계측, 관찰, 검체 분석 등의 방법으로 수행되었다. 본 연구는 2017 년 조사에 참여한 총 8127 명의 대상자 중 신체활동 설문에 응답한 만 19 세 이상의 성인 총 5820 명을

대상으로 실시되었다. 국민건강 영양조사는 생명윤리법 제 2 조 제 1 호 및 동법 시행규칙 제 2 조 제 2 항 제 1 호에 따라 국가가 직접 공공복리를 위해 수행하는 연구에 해당하여 2015 년 부터 연구윤리심의위원회 심의를 받지 않고 수행되고 있다.

Physical activity assessment

신체활동량은 세계보건기구 (World Health Organization; WHO)에서 개발한 Global Physical Activity Questionnaire (GPAQ)에 주당 걷기운동 및 근력운동의 운동 횟수를 묻는 항목이 추가된 modified GPAQ 한글판을 이용하여 측정되었다. 유산소 신체활동량은 여가 시간 및 일에서의 신체활동을 모두 사용하였으며, 신체활동 강도에 따른 에너지 소비량을 고려하여 주당 고강도 유산소 활동 시간을 분으로 환산하여 두배로 계산한 후, 주당 중강도 유산소 활동시간 (분) 과 합산하여 전체 주당 중·고강도 유산소 신체활동 시간 (분) 으로 사용하였고, 근력운동은 주당 실천 일수를 사용하였다. 대상자들은 WHO 에서 18~64 세, 65 세 이상 성인과 노인에게 공통적으로 권고하는 유산소 신체활동과 근력운동 실천 지침의 충족여부에 따라 1) 유산소 운동만 충족하는 그룹 (aerobic only: 주당 150 분 이상의 중강도 유산소 활동 또는 75 분 이상의 고강도 유산소 활동 또는 두 가지 활동의 조합), 2) 근력운동만 충족하는 그룹 (resistance only: 주당 2 회 이상의 근력운동), 3) 근력운동과 유산소 운동을 모두 충족하는 그룹 (combined), 4) 근력운동과 유산소 운동을 모두 충족하지 못하는 그룹 (neither) 으로 구분되었다.

Assessment of other variables

대상자들의 인구사회학적 요인, 체력 및 의학적 요인, 생활습관 요인, 정신건강 요인에 따라 신체활동 충족여부를 분석하였다. 인구사회학적 요인에는 성별, 나이, 결혼상태, 소득수준, 거주지역, 교육수준, 직업, 경제활동 상태, 직장내 직위, 정규직 여부가 변인으로 포함되었으며,

체력 및 의학적 요인에는 만성질환 유병여부, 암 유병여부, 체중, 허리둘레, 체질량지수, 혈압, 공복혈당, 총 콜레스테롤, 고밀도 지단백 콜레스테롤, 중성지방, 악력 (주로 사용하는 손의 3 회 측정 평균) [20], 안정시 심박수, 만성질환 가족력이 포함되었다. 생활습관 요인으로는 흡연 여부, 음주 여부, 수면시간, 아침식사 빈도, 하루평균 칼로리, 수분, 탄수화물, 단백질, 지방 섭취량이 포함되었다. 마지막으로 정신건강 요인으로는 스트레스 인지, 2 주 이상 우울경험 여부, 현재 우울증 유병 여부, 1 년내 자살 생각 경험, 정신상담 경험, 건강관련 삶의 질 다섯가지 영역 (운동능력, 자기관리, 일상활동, 통증/불편, 불안/우울) 이 포함되었다.

Statistical analyses

본 연구에 포함된 모든 통계분석 방법은 복합표본 통계분석 방법을 사용하였다. 여러 가지 변인들 중 명목형 변수를 포함하는 변인과 신체활동 지침 충족여부의 관계분석에는 복합표본 교차분석 (Complex Samples Crosstabs) 이, 연속형 변수를 포함하는 변인과 신체활동 지침 충족여부와의 관계분석에는 복합표본 일반 선형 모델(Complex Samples General Linear Model: CSGLM)이 사용되었다. 또한, 각 변인의 그룹간 중·고강도 신체활동 시간 및 근력운동 빈도의 차이분석에도 복합표본 일반 선형 모델(Complex Samples General Linear Model: CSGLM)이 사용되었다.명목형 변수들 중 그룹간 분포가 고르지 못한 경우, 그룹을 재설정하여 분석하였다 (예: 결혼상태: 미혼 vs. 결혼 후 동거 vs. 결혼 후 별거/사별/이혼 ; 건강관련 삶의 질: 문제없음 vs. 문제 있음). 모든 통계분석은 SPSS 25 를 이용하였다.

RESULTS

Table 1 은 대상자들의 인구사회학적 변인에 따른 신체활동 지침 충족여부를 나타낸다. 전체 대상자 중 Neither 그룹에 포함된 대상자의 수가 가장 많았으며 (66.0%), 그 다음으로는 Resistance only (15.7%), Combined (9.8%), Aerobic only (8.5%) 순이었다.

대상자들의 신체활동 지침 충족여부는 인구사회학적 변인들 중 성별 ($p<0.001$), 나이 ($p<0.001$), 결혼상태 ($p<0.001$), 가구 총 수입 ($p<0.001$), 교육수준 ($p<0.001$), 직업 ($p<0.001$), 정규직 여부 ($p=0.010$)와 통계적으로 유의한 관계를 나타냈다. 특히, 여자가, 나이가 65 세 이상인 경우, 결혼 후 배우자와 따로 사는 경우 (이혼, 사별, 별거), 수입이 가장 낮은 경우, 교육수준이 가장 낮은 경우, 단순노무 근로자인 경우 중·고강도 유산소 신체활동과 근력운동 실천량이 가장 낮은 것으로 나타났고, 비정규직 근로자는 정규직 근로자에 비해 중·고강도 유산소 신체활동량만 낮은 것으로 나타났다.

Table 2 는 한국 성인의 체력 및 의학적 변인들에 따른 신체활동 지침 충족여부를 보여주고 있다. 신체활동 지침 충족여부는 체력 및 의학적 변인들 중 만성질환 여부 ($p<0.001$), 체중 ($p<0.001$), 허리둘레($p=0.005$), 체질량지수 ($p=0.001$), 이완기 혈압 ($p<0.001$), 공복혈당 ($p<0.001$), 고밀도 지단백 콜레스테롤(HDL) ($p=0.001$), 중성지방 (TG) ($p<0.001$), 악력 ($p<0.001$), 안정시 심박수 ($p<0.001$), 만성질환 가족력 ($p=0.001$)과 유의한 관계가 있는 것으로 나타났다. 중·고강도 유산소 신체활동량은 만성질환을 2 개이상 갖고 있는 그룹, 암을 앓고 있는 그룹, 허리둘레가 남자 90cm 이상, 여자 80cm 이상인 경우 그렇지 않은 그룹들에 비해 낮게 나타났다. 반대로 체질량 지수는 25kg/m^2 이상인 그룹이 저체중인 그룹에 비해, 만성질환 가족력이 있는 그룹이 없는 그룹에 비해 중·고강도 유산소 신체활동량이 더 높게 나타났다. 또한, 허리둘레가 남자 90cm 이상, 여자 80cm 이상인 경우, 또는 체질량 지수가 25kg/m^2 이상인 경우가 정상이나 과체중인 그룹보다 근력운동 실천일수가 낮은 것으로 나타났다.

Table 3 는 대상자들의 생활습관과 신체활동 지침 충족과의 관계를 보여주고 있다. 음주빈도 ($p<0.001$), 하루 총 열량 ($p<0.001$), 수분 ($p<0.001$), 단백질($p<0.001$), 지방 섭취량 ($p<0.001$)과 신체활동 지침 충족여부가 유의한 관계가 있는 것으로 나타났다. 주당 2 회 이상

Volume: 41, Article ID: e2019047
https://doi.org/10.4178/epih.e2019047

음주하는 사람이 최근 1 년 내에 음주를 전혀 하지 않은 사람들보다 중·고강도 유산소 신체활동 및 근력 운동량이 더 높은 것으로 나타났다.

Table 4 는 대상자들의 정신건강 및 건강관련 삶의 질 관련 변인들과 신체활동 지침 충족과의 관계를 나타낸다. 스트레스 인지정도 ($p=0.015$), 2 주 이상 우울감 여부 ($p<0.001$), 1 년 내 자살 생각 경험 ($p=0.026$), 운동능력 ($p<0.001$), 자기관리 ($p<0.001$), 일상생활 ($p<0.001$), 통증/불편 ($p<0.001$), 불안/우울 ($p=0.002$) 에 대한 삶의 질이 신체활동 지침 충족여부와 유의한 관계를 보였다. 또한, 스트레스를 가장 많이 받는 그룹, 2 주 이상 우울감이 있었던 그룹, 현재 우울증이 앓고 있는 그룹, 최근 1 년 내에 자살 생각을 했던 그룹, 운동능력, 자기관리, 통증/불편, 불안/우울과 관련하여 조금이라도 문제가 있는 경우에, 그렇지 않은 대상자들보다 중·고강도 유산소 신체활동량이 적은 것으로 나타났다. 근력운동 실천율에서는 스트레스를 가장 많이 받는 그룹, 2 주 이상 우울감이 있었던 그룹, 최근 1 년 내에 자살 생각을 했던 그룹, 운동능력, 자기관리, 일상생활, 통증/불편, 불안/우울과 관련하여 조금이라도 문제가 있는 경우에, 그렇지 않은 대상자들보다 낮게 나타났다.

DISCUSSION

본 연구는 한국 성인들의 신체활동 지침 충족여부를 인구사회학적, 체력 및 의학적, 생활습관, 정신건강 변인에 따라 분석했다. 연구결과, 한국 성인들의 신체활동 실천과 관련하여 몇 가지 특징들을 확인할 수 있었다. 그 첫번째로 한국 성인들의 신체활동 지침 충족률은 매우 낮은 것으로 나타났다. 유산소운동, 근력운동 중 어느 한가지 지침도 충족하지 못하는 사람들의 비율은 전체 대상자의 66.0%에 달했다. 양윤준의 연구에 따르면 불충분한 신체활동을 하는 한국 성인의 비율은 2008 년 24.6%였던 것이, 지속적으로 증가해 2014 년에는 42.9%에 달했다 [21]. 이는 2012 년 보고된 전세계의 비활동적인 성인의 비율인 31.1% 보다도 더 높은 수준이다 [22]. 신체활동이 신체적, 정신적 건강 증진에 미치는 여러 가지 긍정적인

영향을 고려하면 [1, 2] 국가차원에서 신체활동을 장려하고, 신체활동을 늘릴 수 있는 환경을 조성하기 위한 노력이 시급하다. 한국 국민들의 신체활동 실천량이 증가된다면 국민의 건강증진 뿐 아니라 의료비 감소에도 큰 이득이 있을 것으로 기대된다.

두번째로, 한국 성인들의 신체활동량은 건강불평등 요인에 따라 확연히 차이가 났다. 여성이며, 나이가 많고, 소득이 적고, 교육수준이 낮은 경우, 즉, 사회적 약자 또는 저소득층에 해당하는 사람들은 신체활동 지침을 충족하는 비율이 상대적으로 낮은 것으로 나타났다. 이러한 결과는 생리학적 요인 뿐 아니라, 사회적, 환경적인 요인들이 건강행동에 큰 영향을 미친다는 것을 보여주고 있다. 이러한 사회적, 환경적 요인들에 의한 신체활동의 차이는 건강불평등의 악순환을 야기할 수 있다. 불충분한 신체활동은 만성질환 발병, 신체적, 정신적 건강악화라는 결과를 낳고, 질병에 의한 의료비의 증가, 제한된 경제활동은 또 다시 신체활동 감소와 건강불평등을 야기할 수 있다. 이러한 악순환의 고리를 끊기 위해서는 학교나 보건소를 통한 신체활동에 대한 무상교육, 미디어를 이용한 신체활동 실천 홍보와 캠페인 등 실질적이고, 구체적인 대안들이 필요할 것이다.

셋째로, 만성질환의 위험이 높고, 정신건강이 좋지 않은 대상자들은 신체활동이 가장 필요한 집단임에도 불구하고, 신체활동 실천율이 건강한 사람들에 비해 낮은 것으로 나타났다. 본 연구는 단면연구이기 때문에 신체활동과 변인들 사이에 원인과 결과의 관계를 파악할 수 없다. 다시 말해, 신체적, 정신적 건강 악화가 낮은 신체활동을 야기했을 가능성과 동시에 낮은 신체활동이 신체적, 정신적 건강의 악화를 야기했을 가능성도 있다. 하지만 본 연구결과는 많은 선행연구에서 이미 보고한대로 신체활동이 여러 가지 만성질환 [23, 24], 정신건강 [25, 26] 과 매우 밀접한 관계가 있다는 것을 다시 한번 확인할 수 있었다. 의료기관에서 신체활동의 중요성을 더욱 강조하고, 이들에게 올바른 신체활동량과 방법을 권고하는 등 전문 의료인들의 노력이 더해진다면 한국 성인들의 신체활동 실천량을 증가시키는 데 큰 도움이 될 것이다.

추가적으로 본 연구결과는 비만인 그룹 뿐 아니라, 저체중인 대상자들의 신체활동량도 낮은 것을 보여주었다. 선행연구들은 저체중인 사람들은 비만인 사람과 더불어, 정상 체중에 비해 사망률이 높다고 보고하고 있다 [27, 28]. 요즘 한국에서는 마른 몸을 선호하는 경향으로 인해 과도한 다이어트를 시도하고, 올바르지 못한 방법으로 무조건 체중을 감소하려는 사람들이 많다. 건강유지를 위해서 신체활동 참여를 늘리기 위한 노력과 동시에 건강체중 유지를 위한 노력도 고려되어야 할 것이다.

또한, 본 연구결과는 많은 선행 연구들에서 이미 보고한 바와 같이 [29, 30] 체력요인이 신체활동 참여와 관련이 있음을 보여준다. 국민건강 영양조사에서 검진조사 항목에 포함된 체력요인은 악력 한가지 뿐이다. 폐기능과 관련된 변인들이 있기는 하지만 심폐체력을 대표하는 변인으로 보기에는 무리가 있을 뿐 아니라, 결측값이 많아 본 연구에 사용할 수 없었다. 본 연구에서 또 다른 체력 관련 요인으로 사용된 안정시 심박수는 유산소 운동과 직접적인 관련이 있다. 나이와 체력수준을 고려하지 않은 상태에서 안정시 심박수를 유산소 체력 변인으로 간주하는 것은 무리가 있을 수 있겠지만, 유산소 운동을 통해 1 회 심박출량이 증가하고, 안정시 심박수가 감소한다는 것을 고려하면, 안정시 심박수를 통해 유산소 체력을 간접적으로 예측하는 것이 불가능 한 것은 아니다. 더구나 안정시 심박수는 당뇨, 심혈관 질환, 대사증후군 등 여러 가지 만성질환 및 건강관련 요인들과 깊은 연관성을 보여주고 있어 [31-33], 그 자체가 중요한 건강변인으로써의 역할을 할 수 있다. 체력이 여러가지 건강위험요소들과 깊은 관계에 있는 만큼 국민건강영양조사에서 유산소 체력을 조사하는 항목이 추가된다면, 한국 국민들의 신체적 건강을 관찰하고 연구하는데 큰 도움이 될 것이다.

이 외에도 만성질환 가족력, 음주, 식이 관련 변인들도 신체활동 실천률과 연관성이 있음이 확인되었다. 하지만 예상했던 것과는 반대로 만성질환 가족력이 있고, 음주량이 높으며, 칼로리 섭취가 많은 집단에서 더 많은 신체활동을 하는 것으로 나타났다. 이러한 결과는 만성질환 가족력에 의한 운동 동기부여, 남자가 여자보다 음주량이 많다는 점, 한국인의

전체적인 에너지 섭취량이 그리 높지 않다는 점을 고려하면 일부 설명이 가능하다. 하지만 이러한 변인들과 신체활동 실천과의 명확한 결과해석을 위해서는 추가적인 연구 및 다양한 통계방법을 이용한 추가 분석이 필요하다.

본 연구는 단면연구, 제한된 변수 (체력 및 의학적 변수)의 사용 이라는 제한점을 갖고 있다. 하지만 많은 연구대상자, 다양한 항목의 변인들과의 관계분석, 유산소와 근력운동 지침의 구분, 무엇보다도 최근(2017 년) 한국 성인들의 전반적인 신체활동 실천률을 보여주고 있어, 신체활동 연구의 기초 자료로써의 역할을 할 수 있다는 점에서 큰 의미가 있다.

결과적으로, 본 연구는 한국 성인들의 신체활동 실천률를 다양한 변인들과의 관계를 통해 조사하였으며, 그 결과 한국 성인들의 신체활동 실천률이 매우 낮은 것으로 나타났다. 또한, 신체활동 참여가 나이, 성별, 생활습관, 신체적 건강요인 등 생리학적 요인 뿐 아니라 건강불평등 요인을 포함한 여러가지 사회적, 환경적 요인들과 관련이 있음을 확인하였다. 한국 국민들의 건강증진을 위해 신체활동 실천을 늘리기 위한 정부, 교육기관, 의료기관 등의 다양한 노력은 물론 스스로 신체활동의 중요성을 인식하고 실천하는 국민 개개인의 노력이 필요하다.

CONFLICT OF INTEREST

본 논문의 저자는 어떠한 이해관계도 없음을 밝힙니다.

REFERENCES

1. Sallis RE. Exercise is medicine and physicians need to prescribe it! Br J Sports Med 2009;43:3-4.

2. Pedersen BK, Saltin B. Exercise as medicine - evidence for prescribing exercise as therapy in 26 different chronic diseases. Scand J Med Sci Sports 2015;25 Suppl 3:1-72.

3. World Health Organization. Global action plan for the prevention and control of noncommunicable diseases 2013-2020; 2013 [cited 2019 Sep 1]. Available from: https://apps.who.int/iris/handle/10665/94384.

4. Haskell WL, Lee IM, Pate RR, Powell KE, Blair SN, Franklin BA, et al. Physical activity and public health: updated recommendation for adults from the American College of Sports Medicine and the American Heart Association. Med Sci Sports Exerc 2007; 39:1423-1434.

5. Nelson, ME, Haskell WL, Kennedy M. The birth of physical activity guidelines for Americans. J Phys Act Health 2008;5:485-487.

6. Tremblay MS, Carson V, Chaput JP, Connor Gorber S, Dinh T, Duggan M, et al. Canadian 24-hour movement guidelines for children and youth: an integration of physical activity, sedentary behaviour, and sleep. Appl Physiol Nutr Metab 2016;41(6 Suppl 3):S311-S327.

7. Egger G, Donovan RJ, Giles-Corti B, Bull F, Swinburn B. Developing national physical activity guidelines for Australians. Aust N Z J Public Health 2001;25:561-563.

8. Kahlmeier S, Wijnhoven TM, Alpiger P, Schweizer C, Breda J, Martin BW. National physical activity recommendations: systematic overview and analysis of the situation in European countries. BMC Public Health 2015;15:133.

9. Kahn JA, Huang B, Gillman MW, Field AE, Austin SB, Colditz GA, et al. Patterns and determinants of physical activity in US adolescents. J Adolesc Health 2008;42:369-377.

10. Troiano RP, Berrigan D, Dodd KW, Mâsse LC, Tilert T, McDowell M. Physical activity in the United States measured by accelerometer. Med Sci Sports Exerc 2008;40:181-188.

11. Dowda M, Ainsworth BE, Addy CL, Saunders R, Riner W. Correlates of physical activity among U.S. young adults, 18 to 30 years of age, from NHANES III. Ann Behav Med 2003;26:15-23.

12. Juneau CE, Potvin L. Trends in leisure-, transport-, and work-related physical activity in Canada 1994-2005. Prev Med 2010;51: 384-386.

13. Colley RC, Garriguet D, Janssen I, Craig CL, Clarke J, Tremblay MS. Physical activity of Canadian adults: accelerometer results from the 2007 to 2009 Canadian Health Measures Survey. Health Rep 2011;22:7-14.

14. King AC, Castro C, Wilcox S, Eyler AA, Sallis JF, Brownson RC. Personal and environmental factors associated with physical inactivity among different racial-ethnic groups of U.S. middle-aged and older-aged women. Health Psychol 2000;19:354-364.

15. Humpel N, Owen N, Leslie E. Environmental factors associated with adults' participation in physical activity: a review. Am J Prev Med 2002;22:188-199.

16. Crespo CJ, Smit E, Andersen RE, Carter-Pokras O, Ainsworth BE. Race/ethnicity, social class and their relation to physical inactivity during leisure time: results from the Third National Health and Nutrition Examination Survey, 1988-1994. Am J Prev Med 2000;18:46-53.

17. Marshall SJ, Jones DA, Ainsworth BE, Reis JP, Levy SS, Macera CA. Race/ethnicity, social class, and leisure-time physical inactivity. Med Sci Sports Exerc 2007;39:44-51.

18. Yang YJ , Yoon YS , Oh SW , Lee ES. The amount of physical activity of Korean adults measured from the 2001 Korean National Health and Nutrition Survey. J Korean Acad Fam Med 2005;26: 22-30 (Korean).

19. Yang YJ. Current status of physical activity and policy proposals of the national level. Korean J Health Educ Promot 2016;33:47-54 (Korean).

20. Shiratori AP, Iop Rda R, Borges Júnior NG, Domenech SC, Gevaerd Mda S. Evaluation protocols of hand grip strength in individuals with rheumatoid arthritis: a systematic review. Rev Bras Reumatol 2014;54:140-147.

21. Hallal PC, Andersen LB, Bull FC, Guthold R, Haskell W, Ekelund U, et al. Global physical activity levels: surveillance progress, pitfalls, and prospects. Lancet 2012;380:247-257.

22. Warburton DE, Bredin SS. Health benefits of physical activity: a systematic review of current systematic reviews. Curr Opin Cardiol 2017;32:541-556.

23. Kruk J. Physical activity in the prevention of the most frequent chronic diseases: an analysis of the recent evidence. Asian Pac J Cancer Prev 2007;8:325-338.

24. Biddle SJ, Asare M. Physical activity and mental health in children and adolescents: a review of reviews. Br J Sports Med 2011;45:886-895.

25. Penedo FJ, Dahn JR. Exercise and well-being: a review of mental and physical health benefits associated with physical activity. Curr Opin Psychiatry 2005;18:189-193.

26. Katzmarzyk PT, Craig CL, Bouchard C. Original article underweight, overweight and obesity: relationships with mortality in the 13-year follow-up of the Canada Fitness Survey. J Clin Epidemiol 2001;54:916-920.

27. Roh L, Braun J, Chiolero A, Bopp M, Rohrmann S, Faeh D, et al. Mortality risk associated with underweight: a census-linked cohort of 31,578 individuals with up to 32 years of follow-up. BMC Public Health 2014;14:371.

28. Seco J, Abecia LC, Echevarría E, Barbero I, Torres-Unda J, Rodriguez V, et al. A long-term physical activity training program increases strength and flexibility, and improves balance in older adults. Rehabil Nurs 2013;38:37-47.

29. Lim HJ, Seo SM. Physical fitness according to body fat and physical activity levels in women university students. J Korean Soc Living Environ Sys 2010;17:557-562 (Korean).

30. Park H, Lee J, Kim JY, Kim DI, Jeon JY. Relationship between resting heart rate and prevalence of metabolic syndrome and type 2 diabetes mellitus in Korean adults: the fifth Korea National Health and Nutrition Examination Survey (2012). Korean J Obes 2015; 24:166-174 (Korean).

31. Greenland P, Daviglus ML, Dyer AR, Liu K, Huang CF, Goldberger JJ, et al. Resting heart rate is a risk factor for cardiovascular and noncardiovascular mortality: the Chicago Heart Association Detection Project in Industry. Am J Epidemiol 1999;149:853-862.

32. Kristal-Boneh E, Silber H, Harari G, Froom P. The association of resting heart rate with cardiovascular, cancer and all-cause mortality. Eight year follow-up of 3527 male Israeli employees (the CORDIS Study). Eur Heart J 2000;21:116-124.
